# Seasonal Variations in Dietary Diversity and Nutrient Intakes of Women and Their Children (6–23 Months) in Western Kenya

**DOI:** 10.3389/fnut.2021.636872

**Published:** 2021-03-08

**Authors:** Lydiah M. Waswa, Irmgard Jordan, Michael B. Krawinkel, Gudrun B. Keding

**Affiliations:** ^1^Department of Human Nutrition, Faculty of Health Sciences, Egerton University, Egerton, Kenya; ^2^Center for international Development and Environmental Research, Justus Liebig University Giessen, Giessen, Germany; ^3^Institute of Nutritional Sciences-International Nutrition, Justus Liebig University Giessen, Giessen, Germany; ^4^Division of Quality of Plant Products, Department of Crop Sciences, Faculty of Agricultural Sciences, George August University Goettingen, Goettingen, Germany

**Keywords:** seasonal variations, dietary diversity, dietary diversity scores, food groups, nutrient intake

## Abstract

Seasonal variations in food availability and access contributes to inadequate nutrient intakes, particularly in low income countries. This study assessed the effect of seasonality on dietary diversity (DD) and nutrient intakes of women and children aged 6–23 months in a rural setting in Western Kenya. A longitudinal study was conducted among 426 mother-child pairs during the harvest and post-harvest seasons in 2012. Dietary intakes were assessed using 24-h dietary recalls and dietary diversity scores (DDS) and nutrient intakes calculated for both seasons. Effect of seasonality on women dietary diversity scores (WDDS) and children's dietary diversity scores (CDDS) were assessed using generalised linear mixed models (GLMM). The proportion of women consuming diets with high DDS (>4 out of 9 food groups) increased from 36.4 to 52.4% between the two seasons, with mean WDDS being significantly higher in November compared to July/August (4.62 ± 1.43 vs. 4.16 ± 1.14, *P* < 0.001). A significantly higher proportion of children consumed foods from ≥4 out of 7 food groups in November compared to July/August (62.4 vs. 52.6%, *P* = 0.004). Mean CDDS (3.91 vs. 3.61, *P* = 0.004) was low but significantly higher in November compared to July/August. Estimated marginal mean WDDS increased from 4.17 to 4.38, and decreased for CDDS from 3.73 to 3.60 between the seasons. Seasonality had a small but significant effect on WDDS, *P* = 0.008 but not on CDDS, *P* = 0.293. Increase in CDDS in November was due to age and not seasonal effect. Higher women education and household food security were associated with higher WDDS and CDDS. Intakes of iron, calcium and vitamin E were higher among women in November and significantly different between the seasons. Agro-ecological zone, ethnic group and home gardening influenced nutrient intakes of the women. Seasonality had an effect on the DD of women but not of children, thus other factors apart from food availability influence the quality of children's diets during the complementary feeding period. With increasing age and transition to family foods, children's DD is expected to be affected by seasonality. Integrated interventions to alleviate seasonal food insecurity and strengthen rural households' resilience against seasonal deterioration in diet quality are recommended.

## Introduction

Malnutrition in all its forms including undernutrition, micronutrient deficiencies, and the emerging problem of overweight and obesity, and the resulting diet-related non-communicable diseases remains a challenge globally. Estimates from FAO indicate that the number of hungry people in the world has increased in the past decade to more than 820 million in 2018. Africa is the region with the highest prevalence of undernourishment in the world with an estimated 256.1 million hungry people (1). According to the World Health Organisation (WHO), 462 million adults are underweight, while 1.9 billion are overweight and/or obese (2). An estimated 144 million, 47 million and 38.3 million children under 5 years in the world are stunted, wasted, and overweight/obese, respectively. Africa bears the second greatest share of all forms of malnutrition after Asia with 40, 27, and 24% of all stunted, wasted and overweight children under 5 years (3). These estimates, do not however capture the even more widespread problem of micronutrient deficiencies, which affects over 2 billion people in the world (4). The deficiencies of vitamin A, iron, iodine and zinc represent a major threat, particularly to young children and pregnant women in low-income countries. An estimated one third of the developing world's children under the age of 5 are vitamin A deficient while anaemia prevalence in girls and women of reproductive age remains high at 32.8%, with iron deficiency anaemia during pregnancy accounting for one fifth of all maternal deaths (4, 5). According to the 2011 Kenya National Micronutrient Survey Report the prevalence of anaemia, iron deficiency and iron deficiency anaemia among pregnant women in Kenya was among the highest at 41.6, 36.1, and 26%, while it was 21.9, 21.3, and 14% among non-pregnant women, respectively (6). Similarly, estimates from the same report showed that pre-school children had a higher prevalence of anaemia, iron deficiency and iron deficiency anaemia (26.3, 21.8, and 13.3%, respectively), compared with school-age children (16.5, 9.4, and 4.9%, respectively). The prevalence of Vitamin A deficiency (VAD) was even highest among pre-school children (9.2%) compared with all other groups. A smaller study also confirmed the overall burden of anaemia, iron deficiency and VAD among primary school children residing in urban and rural areas (38.9 vs. 28.6%; 2.9 vs. 14.3%, and 14.7 vs. 8.6%), respectively (7). This study concluded that the nutritional and micronutrient status of urban school children with moderate anaemia was better than in a rural area in Eastern Kenya. This shows that there is still a gap in identifying solutions to combat micronutrient deficiencies among populations residing in rural areas. Fortunately estimates from the 2014 Kenya Demographic and Health Survey (KDHS) indicated that the prevalence of stunting, wasting and underweight among children aged below 5 years had dropped from 35, 7, and 16% in 2008–2009 to 26, 4, and 11% in 2014, respectively (8). Similarly, while the percentage of women (15–49 years) who were thin (BMI < 18.5 kg/m^2^) declined slightly from 12 to 9%, the proportion of those who were overweight (BMI ≥ 25 kg/m^2^) and obese (BMI ≥ 30 kg/m^2^) increased from 25% in 2008–2009 to 33% in 2014.

Malnutrition, particularly micronutrient deficiencies are attributed to many factors and can still occur even when there is adequate food to meet the energy requirements among different population groups (9). Many rural households in resource poor settings subsist on staple-based diets with few or no animal products, fruits and vegetables. This consumption of poor quality diets coupled with the high rates of infectious diseases are major factors responsible for the high burden of micronutrient deficiencies and their consequences in these resource poor settings (10, 11).

Seasonality, including variations in temperature and rainfall is a key factor influencing food production, availability and access. This is especially the case among rural households in developing countries which depend on food from their own agricultural production activities and on their annual harvest of staple crops following the main rain season (12–14). Often, many such households also have limited financial resources, and are thus more likely to experience seasonal changes in food access during the lean seasons due to high food prices (15–17). Seasonal variations in food availability leads households to adapt their food consumption patterns by modifying not only the number of meals and quantities of foods they consume, but also the types and quality of foods they consume (18). In addition to affecting food availability and access, the food shortage period is also often characterised by increased agricultural workload and morbidity, which coupled with inadequate nutrient intake contribute to poor health and nutritional status (13, 14). Seasonal variations in food availability and access contributes to reduced food security and DD (12, 19–21) and consequently to inadequate intake of energy and essential nutrients, particularly micronutrients (22–24) which are linked to negative consequences on health and nutritional status (9, 11, 25).

Women of child bearing age, especially during pregnancy and lactation, and infants and young children who experience rapid growth and are prone to suffer from infectious diseases are particularly vulnerable to suffer from malnutrition due to their increased nutrient requirements (26, 27). The adverse short- and long-term consequences of inadequate energy and nutrient intakes especially during pregnancy, lactation, and early childhood have been well-documented (28, 29). Consumption of diversified diets, including a variety of animal source foods, fruits and vegetables, is therefore recommended for women of reproductive age and young children at all times in order to support normal growth and good health (30).

Studies conducted in low income countries have provided evidence of the effect of seasonality on food/ nutrient intake and DD of households (31, 32) and for different population groups including older children aged above 2 years (15, 33), school going children (17, 34) and women (12, 13, 21, 35). While most of these studies assessed the influence of seasonality on single population groups, less studied is the influence of seasonality on the dietary intakes of mothers and their young children aged 6–23 months. This study aimed to examine seasonal variations in DDS, food and nutrient intakes among women and their children aged 6–23 months during two seasons in a rural setting in Western Kenya. The effect of seasonality and other factors on the DD and nutrient intakes of women and children were also investigated. Additionally, we assessed the relationship between the women's and children's DD. This study was embedded in a larger project entitled, “Improving nutritional health of women and children through increased utilisation of local agro-biodiversity in Kenya,” (INULA). The project was implemented by Biodiversity International, Nairobi, Kenya in collaboration with the Institute of Nutritional Sciences, Justus Liebig University-Giessen, Germany.

## Materials and Methods

### Study Area and Population

The study was conducted in 4 sub-counties (formerly districts) in rural Western Kenya with different characteristics as described in Table 1. The majority of the population in the study area is involved in agriculture with subsistence farming as the main economic activity. Fishing is also practised in Bondo Sub-County which is located along the shores of Lake Victoria.

**Table 1:** Characteristics of the study areas in Western Kenya.

**District/Sub-county**	**Bondo**	**Mumias**	**Teso south**	**Vihiga**
Main ethnic group	Luo	Luhya	Teso	Luhya
Agro-ecological zones^a^	LM3, LM4, and LM5	LM1	LM1 and LM2	UM1
Population density^b^	266	609	460	1,101
Annual mean rainfall^b^	1,020–1,100 mm	1,800–2,000 mm	1,550–1,800 mm	2,000 mm
Annual mean temperature	22.0–22.7°C	21.0–22.0°C	21.4–22.3°C	18.5–21.0°C

*LM3, semi-humid lower midland zones; LM4, transitional lower midland zones; LM5, semi-arid lower midland zones; LM1, humid lower midland zones; LM2, sub-humid lower midland zones; UM, humid upper midland zones*.

a *Kenya National Bureau of Statistics (36)*.

b *Jaetzold (37)*.

### Study Design and Sample

**Figure 1:**
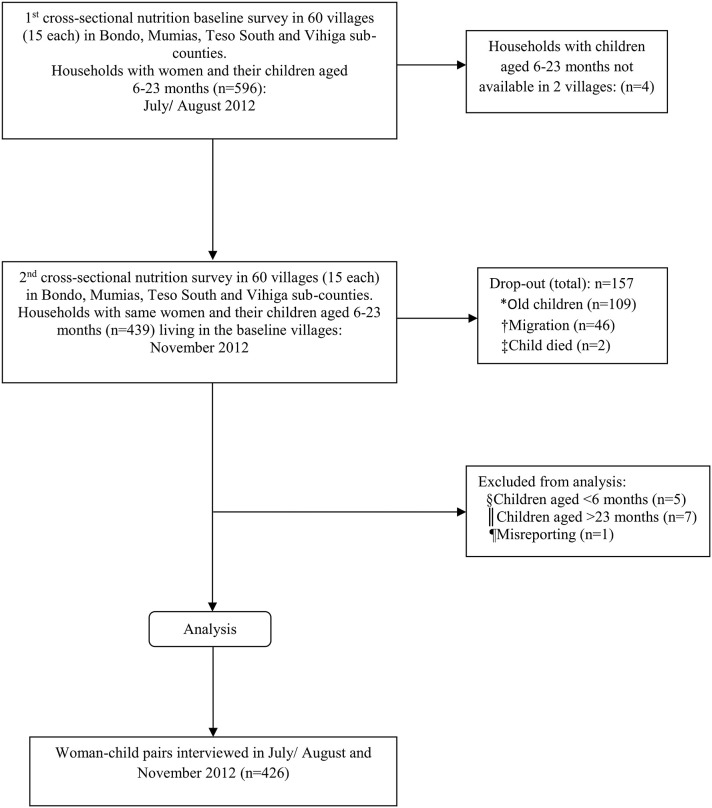
Study profile. *old children: the children were aged above the required 23 months; †migration: sample households relocated from study area; ‡child died: the sampled children had passed away; §child < 6 months: age of children less than the required age of 6 months; ||age of children above the required age of 23 months; ¶Misreporting: Missing data.

The sample for this longitudinal study stemmed from 2 cross-sectional nutritional surveys that were carried out in July/August 2012 (harvest season) and November 2012 (post-harvest season, which also coincided with the short-rain season). At baseline (July/August), a two-stage cluster sampling technique was applied. First, 60 villages (15 per sub-county) were randomly selected with a probability proportional to size (PPS) method using open source software R. Secondly, 10 households with women (caregivers) and their children aged 6–23 months residing in the sampled villages were randomly selected from household lists prepared by community health workers of the respective sampled villages. In households where more than one eligible woman was present, the woman with the youngest child who would still be aged below 2 years at the time of the survey in November was selected and included in the study. A total of 596 women-children pairs were interviewed in July/August. The same women-children pairs interviewed in July/August and whose children were still aged between 6 and 23 months were interviewed again in November to capture a different season. A total of 439 women-children pairs participated in both surveys (panel), while 157 were lost at follow-up in November. The main reasons for drop out included: the children having grown older than the eligible age of 23 months (*n* = 109), migration of sampled household from study area (*n* = 46), and death of the index child (*n* = 2). Thirteen women-children pairs were excluded from analysis since the children were aged either below 6 months (*n* = 5) or above 23 months (*n* = 7), and 1 case (*n* = 1) for misreporting. This resulted in a sub-sample of 426 women-children pairs that formed the panel data used in the analysis within the present study. The study profile is presented in Figure 1.

During the baseline survey, the sample size was calculated based on the estimated prevalence of children aged 6–23 month with a DDS ≥ 3 food groups (for breastfed) and ≥4 food groups (for non-breastfed) children in the project area (49.7%) (38), with a confidence level at 95% (standard value of 1.96), margin of error at 5% (standard value of 0.05), and design effect of 1.5 since there was no previous information on the design effect in the project area. This resulted in a minimum required sample size of 576, which was further increased by 5% to 605 to account for contingencies. This figure was rounded off to 600, a number that matched well with the 4 sub-counties sampled for the study (150 households per sub-county).

In the statistical model that was set up to test the main hypothesis—in this case seasonal differences in WDDS as a main effect, including covariates (wealth index, ethnic group, household size, education of woman (years), age of women (years), household hunger score (HHS), home gardening, agro-ecological zone and sub-county), and including the interaction survey by ethnic group–the realised sample size of 414 women (828 observations at both time points) was able to detect a least significant difference (LSD) of 0.15 in WDDS. This represents a rather small difference in WDDS. Since small and not necessarily meaningful effects might reach statistical significance, we are not only interpreting the *P*-values but the effect sizes as well.

All the women included in the study gave their verbal and informed written consent to participate in the study. Research permission including ethical approval for this study was obtained from the National Council of Science and Technology (NCST) Nairobi, Kenya.

### Data Collection

Data was collected by a team of 8 trained enumerators with Bachelor of Science degrees in Nutrition, and who were conversant in English, Kiswahili, and the local languages spoken in the study area (Luhya, Luo, and Teso). Pre-tested semi-structured questionnaires were used to collect data through face to face interviews with the women in their homes.

#### Socio-Economic Status

Socio-economic data as well as data on water and sanitation were collected at both the household and individual level. Variables on the ownership of valuable durable assets, housing characteristics, source of drinking water, type of sanitation facilities, and land ownership were used to construct the household wealth index score. Using principal component analysis (PCA), weights were assigned to each variable in the household; the weighted scores for each household were then summed up to come up with the wealth index score with a high score meaning high wealth (39).

#### Food Consumption

The 24-h dietary recall method was used to assess the food consumption patterns at household and individual levels for the women and their children during the individual interviews conducted during each survey (40). At the household level, the women who are responsible for food preparation were asked by the trained enumerators to recall all the foods that they or any member of their households had eaten at home during the previous 24 h. Only foods consumed at home, and not those purchased and consumed outside the home during the previous 24-h were recorded during the qualitative 24-h dietary recalls conducted at household level (41). One quantitative 24-h dietary recall was conducted at individual level and separately for the women and children during the two seasons. The women were asked to describe all the foods and drinks that they or their children had eaten or drank 24 h preceding each of the 2 surveys. All the foods that the women and their children had consumed at home and those purchased and consumed outside the home were recorded. The names and where possible ingredients of all the dishes, snacks, beverages, or any other foods that they or their children had consumed at home or outside the home were recorded. The amounts of all the foods and beverages consumed by the women and the children were estimated using local household measures such as cups, plates and spoons. The exact amount of each ingredient in mixed dishes such as relish of different vegetables was collected before the survey from few households from each sub-county and standard recipes calculated.

The information collected from the 24-h recalls was used to assess DD, which is defined as the count of individual food items or food groups consumed over a given period of time, usually a reference period of the previous 24 h (30). DD is a qualitative measure of food consumption that can be assessed at either household or individual level by counting the number of food groups consumed and then calculating the DDS (41). DD has been shown to be a good predictor of the quality and nutrient adequacy of diets for women (35, 42) and children (43, 44), and as proxy indicator of household food security (45, 46). DD has also been positively associated with nutritional status (45–48). DDS were calculated separately for the household, women and children in July/August and November. The household dietary diversity score (HDDS) and WDDS were constructed based on 12 and 9 food groups, respectively, following the recommendation from FAO (41). The HDDS has a sum of 12 scores ranging from 0 to 12 while WDDS ranges from 0 to 9 with a sum of 9 scores. At the time of designing and conducting the current study, the newly developed Minimum Dietary Diversity-Women (MDD-W) Global Dietary Diversity Indicator for women that recommend consumption of at least 5 out of 10 food groups (49) was not available. Thus, the choice of the cut-offs to define the diets of the women in this study as having low, medium or high DDS were defined by terciles based on the observed distribution of the DDS during the survey in July/August. The same cut-offs were applied in November. Women with diets consisting of <4 food groups were defined as having low DDS, 4 food groups as having medium DDS, and >4 food groups as having high DDS. The CDDS was also constructed from the 24-h recall data and based on seven food groups recommended by WHO (50). The CDDS ranges from 0 to 7, and children who consume foods from at least 4 or more food groups are considered to have received the minimum dietary diversity (MDD) (51).

The amount of foods consumed by the women and children 24-h preceding each survey was converted into nutrients using the open source software package, “Nutri-Survey” (52) The Nutri-Survey program was based mainly on the Kenyan food database with addition of missing foods from the Prota database (http://www.prota4u.org/) for some traditional vegetables as well as the German food database (provided by NutriSurvey) and other databases such as FoodData Central (https://fdc.nal.usda.gov/).

We conducted single 24-h dietary recalls during each of the surveys during the 2 different seasons in July/August and November including only usual days in terms of food consumption. Unusual low and high energy intakes were expected among a few women who reported either not having consumed any food or consumed only small quantities of foods the day preceding the survey, mainly due to sickness. On the other hand, unusual high energy intakes were also expected among some women mainly due to festivities such as funerals which were common in the study areas. As a result, these women had unusual low energy intakes during one season compared with the other season when they had normal foods intakes. In order to have a clearer interpretation of the results, with regards to seasonal differences in nutrient intakes, we excluded women with energy intakes <2,092 or >14,644 kJ/day (<500 or >3,500 kcal/day) to control for unrealistic energy under-reporting and over-reporting (53). This represented 5% (*n* = 22) of the women who had either unusual low or high energy intakes during any of the 2 surveys. We decided in favour of this approach as our main aim was to analyse seasonal differences, and for this an extreme energy intake on 1 day due to sickness or a feast—and not due to seasonal food availability—would have disrupted/disturbed the analysis.

Individual energy and nutrient requirements were determined for each woman during each season based on their age and physiological status (pregnancy and lactation) and with reference to the estimated average requirements (EAR) values (54–56). The percentage of women who were pregnant in July/August and November were 4.7 and 6.2%, respectively. Thus, an additional 1,891 kJ/day (452.kcal/day) and 1,674 kJ/day (400 kcal/day) were added for pregnant and lactating women, respectively (55). The mean energy and nutrient intakes were calculated and compared between the two seasons. The prevalence of inadequate nutrient intakes among the women during the two seasons were estimated based on individual requirements and using the EAR reference values (54). In addition, the amount of single food (g/day) consumed by the women were also estimated using this programme and compared between the two seasons.

The estimated requirements for macronutrients and micronutrients from complementary foods for children aged 6–23 months were calculated based on an assumption of average breast milk intake for the age groups 6–8, 9–11, and 12–23 months following the recommendation for developing countries (57). Based on this recommendation, children in the age groups 6–8 and 9–11 months with average breastmilk intake have no additional requirements for folic acid and vitamin C from complementary food. Similarly, children aged 6–23 months with average breast milk intake do not need additional requirements for vitamin B6 from complementary foods. Thus, analysis with regards to folic acid and vitamin C was done only for breastfed children aged 12–23 months and non-breastfed fed children who received complementary foods during the two seasons. Analysis with regards to vitamin B6 was done only for non-breastfed children who received complementary foods during the 2 time points. Hence the different and small n-values for folic acid, vitamin C and vitamin B6 compared with the rest. It is also important to note that the study children had grown older at the time of the second survey in November and thus had different and higher requirements for energy and nutrients from complementary foods. In order to control for age, we determined the children's median percentage met requirements for energy and nutrients from complementary foods in July/August and November. The ‘median percentage met requirements' is the median value for the percentage of requirements that were met for energy and selected nutrients. We also determined the differences in the met percentage requirements for energy and nutrients from complementary foods for the children between the two seasons.

#### Household Food Insecurity Assessment

Household food insecurity (HFI) was measured using the household hunger scale (HHS) (58). The HHS consists of 3 occurrence questions that provide information on the behaviour of households with regard to 3 food conditions related to household food insecurity, insufficient food quality and insufficient intake of food during a 30-day reference period. An affirmative response to each occurrence question is then followed by a frequency-of-occurrence question to determine if the condition happened rarely (1–2 times), sometimes (3–10 times) and often (≥10 times) during the 30 days' reference period. Data from the HHS was used to construct a categorical household hunger scale score (HHS score) indicator with 3 household hunger categories: 0–1 indicating little to no hunger in the household; 2–3, moderate hunger in the household; and 4–6, severe hunger in the household.

### Data Management and Statistical Analyses

Descriptive analyses were performed to provide the background characteristics of the study population. The nutrient intake data for vitamin E and fat were log-transformed to correct for data distribution abnormalities including skewed data and outliers before data analysis. Differences in the proportion of women and children consuming foods from different food groups between the two seasons was assessed using the McNemar test. Differences in intakes of foods (g/day) between the seasons by the women was determined using the sign test, while differences in mean nutrient intakes were assessed using the *t*-test. Wilcoxon sign-rank test was performed to test for difference in the median percentage met requirements for energy and nutrients from complementary foods among the children between the two seasons.

The Bonferroni-Holm correction for multiple comparison tests were performed to correct for type 1 error in the multiple analyses of food groups consumed, food (g/day) and nutrient intakes between the two seasons, and adjusted *P*-values reported (59). This was done in order to ascertain that the observed changes in food and nutrient intakes between the seasons were not by chance. For each test, the *P*-values were sorted in order from the smallest to the largest and the total number of *P*-values (m) determined. Then, the total number of *P*-values, in this case “m” was multiplied by the first smallest *P*-value. If the first *P*-value remained significant, the second *P*-value was multiplied by the total number of *P*-values less one (m−1). This sequential procedure was repeated until the last and largest *P*-value was multiplied by 1.

The assumption of linearity between WDDS and CDDS was checked and found to be reasonable. Pearson's correlation was used to assess the relationship between WDDS and CDDS in July/August and November, as well as the relationship between change in WDDS and change in CDDS between the two seasons. The seasonal effect and the effect of other variables on DDS were assessed separately for the women and children using the generalised linear mixed model (GENLINMIXED) approach, taking into account the repeated measurements, modelling the DDS as count variables with Poisson regression, and adjusting for covariates. Women age (years) and education (years), wealth index, household size, household hunger score, home gardening, ethnic group, agro-ecological zone, and sub-county were used as covariates in the GENLINMIXED model with WDDS. The GENLIMIXED model used to assess the effect of seasonality on CDDS included the covariates age of children (months), wealth index, ethnic group, household size, household hunger score, home gardening, agro-ecological zone, and sub-county. Women's age (years) and education (years) were included as additional covariates in the GENLINMIXED model with CDDS to assessing the effect of other variables on CDDS.

Univariate analysis of variance (UNIANOVA) models, including age of women (years), education of women (years), wealth index, household hunger score, household size, breastfeeding status, home gardening, and agro-ecological zone as covariates were used to determine the factors influencing nutrient intakes among the women. All statistical analyses were performed using IBM SPSS Statistics version 22.0 statistical software program (60).

## Results

The basic characteristics of the study population are presented in Table 2. The average age of the study women was 27.4 years, with 87.1% being married. Most of the women (74.0%) had primary education. The household size ranged from 2 to 17 persons with an average of 6 persons. Approximately one third of the households (29.6%) experienced moderate hunger. The prevalence of underweight and overweight/obese among the study women was 10.3 and 16.7% respectively, while 28.4, 12.9, and 3.5% of the study children were stunted, underweight and wasted, respectively.

**Table 2:** Basic characteristics of study population in Western Kenya (*n* = 426).

**Characteristics**	***n* = 426**	**%**
**Age of women (years)**		
Mean	27.4	
SD	7.9	
**Marital status**		
Married	371	87.1
Widowed/divorced/single	55	12.9
**Maternal education**		
No formal education	27	6.3
Primary	351	74.0
Secondary	74	17.4
Higher	10	2.3
**Age of children (months)**		
Mean	12.78	
SD	4.0	
**Sex of children**		
Male	217	50.9
Female	209	49.1
**Household size**		
Mean	6.06	
SD	2.3	
**Ethnic group**		
Luo	111	26.1
Luhya	232	54.5
Teso	77	18.1
Others	6	1.4
**Household food insecurity**		
Little to no hunger in household	285	66.9
Moderate hunger in household	126	29.6
Severe hunger in household	15	3.5

*SD, standard deviation*.

### Seasonal Variations in Dietary Diversity of Women and Children

**Figure 2:**
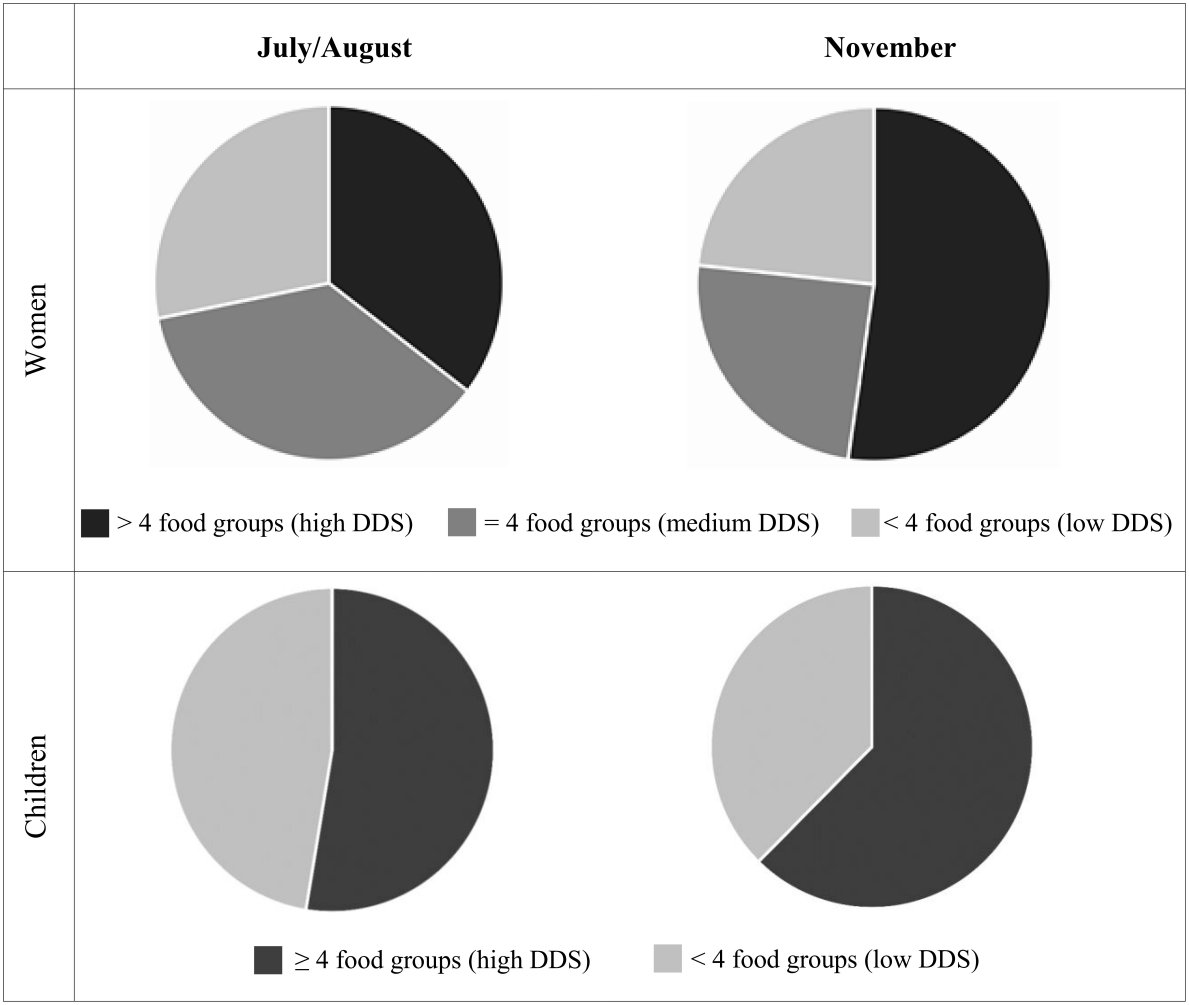
Distribution of Dietary Diversity Scores (DDS) and proportion of women (*n* = 426) who had diets with low (<4 food groups), medium (=4 food groups) and high (>4 food groups) DDS and children (*n* = 426) who consumed diets with high (≥4 food groups) and low (<4 food groups) dietary diversity and in July/August and November 2012.

We observed changes in the food consumption patterns of the women and children between the seasons, with the women and children consuming foods from more food groups in November compared with July/August. There was a significant increase in the proportion of women who consumed dark green leafy vegetables (85.9 vs.73.2%, *P* < 0.001), legumes, nuts and seeds (44.1 vs. 29.8%, *P* < 0.001) and vitamin A rich fruits and vegetables (22.3 vs. 12.9%, *P* = 0.002) in November compared with July/August (Table 3). The distributions of DDS among the women were also found to be different between the two seasons, with the proportion of women consuming diets with high DDS (>4 food groups) increasing from 36.4% in July/August to 52.4% in November (Figure 2). On the other hand, the proportion of women who consumed diets with medium (4 food groups) and low (<4 food groups) DDS decreased from 35.4 to 24.4% and 28.2 to 23.3% between the two seasons, respectively. Consequently, the observed mean WDDS (SD) was significantly higher in November compared to July/August [4.62 (1.43) vs. 4.16 (1.14), *P* < 0.001]. Overall, the diets of nearly all the women included cereals, vegetables, oils/ fats, sugar, and tea during the two seasons. These foods dominated and formed the basic diets of the women with low DDS (<4 food groups). Women with medium DDS (4 food groups) also consumed milk, fish, pulses, and fruits in addition to the cereals, vegetables, oils/fats, sugar, and tea. Next to the foods consumed by women with both low and medium DDS, women with high DDS (>4 food groups) also consumed animal source foods including milk, fish, meat, and eggs during the two seasons.

**Table 3:** Consumption of foods from different food groups by women and children 6–23 months in Western Kenya (*n* = 426).

**Food groups**	**July/August 2012**		**November 2012**		Adj. P^*^
	***n***	**%**	***n***	**%**	
**Consumption of foods from 9 food groups by women^†^**					
Starchy staples (cereals, roots, and tubers)	424	99.5	425	99.8	1.000
Dark green leafy vegetables	312	73.2	366	85.9	<0.001
Vitamin A rich fruits and vegetables	55	12.9	95	22.3	0.002
Other fruits and vegetables	376	88.3	372	87.3	1.000
Organ meats	6	1.4	11	2.6	1.000
Meat and fish	207	48.6	204	47.9	1.000
Eggs	27	6.3	43	10.1	1.000
Legumes, nuts, and Seeds	127	29.8	188	44.1	<0.001
Milk and milk products	239	56.1	265	62.2	0.128
**Consumption of food from seven food groups by children 6–23 months^‡^**					
Grains, roots, and tubers	411	96.5	405	95.1	1.000
Vitamin A rich fruits and vegetables	239	56.1	280	65.7	0.015
Other fruits and vegetables	303	71.1	334	78.4	0.059
Flesh foods	139	32.6	148	34.7	1.000
Eggs	49	11.5	50	11.7	1.000
Legumes, nuts, and seeds	144	33.8	174	40.8	0.153
Dairy products	254	59.6	275	64.6	0.341

Adj., Adjusted

† *Based on 9 food groups FAO (41)*.

‡ *Based on seven food groups WHO (50)*.

* *McNemar test, Adjusted P-value- Bonferroni–Holm correction test for multiple comparisons*.

Similarly, a higher proportion of children consumed foods from more food groups in November compared with July/August. However, after controlling for type 1 error, the observed increase remained significant only for the proportion of children who consumed vitamin A rich fruits and vegetables between the two seasons (65.7 vs. 56.1%, *P* = 0.015). The observed mean CDDS (SD) was also significantly higher in November compared with July/August [3.91 (1.43) vs. 3.61 (1.58), *P* = 0.004]. A significantly higher proportion of children consumed foods from ≥4 out of 7 food groups in November compared with July/August [62.4% vs. 52.6%, *P* = 0.004] (Figure 2). Overall diets of the women and their children were dominated by starchy staples with the consumption of animal source foods, vitamin A rich fruits and vegetables, and legumes, nuts and seeds being notably low during both seasons.

### Seasonal Variations in Food and Nutrient Intakes Among Women and Children

Results with regards to the estimated intakes of foods (g/day), showed a significant decrease in the amounts of cereals [347.19 g (202.78) vs. 300.47 g (151.56), *P* = 0.018] and fruits [62.93 g (146.62) vs. 34.42 g (96.07), *P* = 0.004] consumed by the women in November. On the other hand, we found a significant increase in the amounts of pulses [65.68 g (130.43) vs. 98.06 g (167.56), *P* = 0.009] and milk [124.68 g (136.66) vs. 140.47 g (142.31), *P* = 0.043] consumed by the women in November. However, the observed significant differences in the amounts of cereals, fruits, pulses and milk consumed by the women between the seasons were very small and found to be non-significant after further analysis to control for type 1 error. In general, the amounts of animal source foods consumed by the women were low during both seasons. Tea consumption was notably high among the women in the study area during both seasons.

Energy, protein and fat intakes of the women were slightly higher in November but not significantly different between the seasons (Table 4). Carbohydrate intake was slightly lower in November but not significantly different between the two seasons. Intakes of most micronutrients were slightly higher in November, with the exception of vitamin B_1_, folic acid and phosphorous which were lower, and vitamin B_2_ which was similar across the seasons. After controlling for type 1 error, only the intakes of iron [mean (SD) 14.04 (5.91) and 15.66 (6.16), *P* = 0.001], calcium [mean (SD) 419.62 (240.59) and 500.23 (249.56), *P* <0.001] and vitamin E (median, 25–75 percentile 4.25, 3.03–6.04, and 4.94, 3.47–7.19, *P* = 0.001) were found to be significantly different between the seasons. Except for carbohydrates, magnesium and phosphorus, intakes of energy and most nutrients by the women were less than (or below) the EAR during both seasons.

**Table 4:** Nutrient intakes among women in July/August and November 2012 in Western Kenya (*n* = 404).

**July/August 2012**						**November 2012**					
**Nutrient and unit of measure**	**Mean**			**Mean % met EAR**	**% of respondents <100% of EAR**	**Mean**			**Mean % met EAR**	**% of respondents <100% of EAR**	Adj. P^*^
	**EAR^†^**	**Nutrient intake**				**EAR^†^**	**Nutrient intake**				
		**Mean**	**SD**				**Mean**	**SD**			
Energy, kJ	11,830.66	7,205.63	2,572.98	61.03	94.06	11,628.08	7,241.82	2,366.32	62.52	94.55	1.000
Protein, g	68.52	55.55	25.53	82.63	74.26	65.55	58.65	27.3	92.56	61.88	0.550
Fat, g	–	39.51	25.47	–	–	–	40.07	23.18	–	–	1.000
Carbohydrates, g	152.88	308.9	116.12	205.66	5.45	145.38	301.18	102.32	215.4	3.71	1.000
Vitamin A RE, μg	843.18	761.76	616.64	94.8	59.95	790.47	860.55	616.63	115.85	49.63	0.165
Vitamin E, mg^a^	15.43	4.25	3.03, 6.04	38.15	59.95	14.88	4.94	3.47, 7.19	46.1	49.63	0.010
Vitamin B_1_, mg	1.17	1.76	1.96	151.64	31.68	1.13	1.61	0.84	144.11	31.68	0.148
Vitamin B_2_, mg	1.26	1.12	0.46	90.63	67.33	1.21	1.15	0.43	97.55	59.16	1.000
Vitamin B_6_, mg	1.64	1.72	0.82	107.06	54.7	1.56	1.86	0.84	122.74	40.35	0.096
Folic acid, μg	440.45	374.24	293.72	85.66	67.57	426.06	361.66	304.33	86.69	67.33	1.000
Vitamin C, mg	94.43	80.88	60.97	87.72	68.56	89.23	82.96	62.1	98.16	62.87	1.000
Iron, mg	7.41	14.04	5.91	203.4	10.89	7.83	15.66	6.16	218.12	10.4	0.001
Zinc, mg	10.03	9.6	3.92	97.34	58.66	9.58	10.03	3.93	108	47.28	0.617
Calcium, mg	814.85	419.62	240.59	51.82	93.07	814.85	500.23	249.56	61.77	90.1	<0.001
Magnesium, mg	268.38	481.41	179.28	179.54	8.91	268.9	499.02	165.54	185.6	6.68	0.782
Phosphorus, mg	603.51	1232.38	522.77	207.78	7.18	603.51	1,225.88	493.93	206.51	8.66	0.840

*EAR, estimated average requirements; SD, standard deviation; kJ, kilo joule; g, grammes; μg, micrograms; mg, milligrammes*.

a *Variable log-transformed before analysis, median, 25th-75th percentiles reported*.

† *Mean EARs calculated based on age and physiological status (pregnancy or lactation status) of the women in July/August and November*.

*EARs references: Energy, carbohydrates and protein (55); vitamin A, vitamin B_1_, vitamin B_2_, vitamin C, vitamin E, folic acid, iron, zinc, magnesium, and phosphorus (52), and calcium (56)*.

* *t-test used to determine differences in mean nutrient intakes between July/August and November*.

*Adjusted P-values: Bonferroni–Holm correction test for multiple comparisons*.

Except for iron, zinc, calcium and phosphorus, 50% of the children met more than 100% of their energy, protein, fat, carbohydrates, vitamin B1, vitamin B2, vitamin B6, and folic acid requirements from complementary foods in July/August (Table 5). The proportion of vitamin C, iron and calcium requirements met by the children from complementary foods were <50% in July/August, and thus notably low. The same trend was observed in November, however, the proportion of vitamin C, iron, zinc and calcium requirements met from complementary foods were higher during this season. Except for fat, folic acid, vitamin B6, and vitamin C, the children achieved significantly higher requirements for energy and most nutrients from complementary foods in November compared with July/August.

**Table 5:** Median percentage met requirements for energy and nutrients from complementary foods among children 6–23 months in July/August and November 2012 in Western Kenya.

**Nutrients and unit of measure**	***n***	**July/August 2012**			**November 2012**			Adj P^*^
		**Median percentage met requirements^†^**	**Percentiles**		**Median percentage met requirements^†^**	**Percentiles**		
			**25th**	**75th**		**25th**	**75th**	
Energy (kJ)	426	140.11	88.83	233.84	193.28	125.17	276.49	<0.001
Protein (g)	426	424.75	225.50	797.13	625.41	328.21	999.25	<0.001
Fat (g)	426	216.09	82.19	525.92	270.07	111.27	488.46	0.499
Carbohydrates (g)	426	117.62	77.20	188.60	172.64	112.16	240.93	<0.001
Vitamin B_1_ (mg)	426	290.00	173.75	500.00	420.00	263.75	600.00	<0.001
Vitamin B_2_ (mg)	426	135.00	75.00	230.00	175.00	110.00	270.00	<0.001
Vitamin B_6_ (mg)	52^a^	206.67	125.83	354.17	263.33	146.67	388.33	0.150
Folic acid (μg)	52^b^	220.44	90.03	415.27	279.59	124.08	580.23	0.148
Vitamin C (mg)	67^b^	0.00	0.00	81.92	60.32	3.44	148.84	0.190
Iron (mg)	426	18.08	10.42	27.76	26.08	16.48	38.25	<0.001
Zinc (mg)	426	54.64	34.76	93.04	80.60	52.62	116.00	<0.001
Calcium (mg)	426	36.11	18.70	72.30	50.13	27.43	83.88	<0.001
Magnesium (mg)	426	233.94	147.32	367.70	337.81	216.54	449.08	<0.001
Phosphorus (mg)	426	88.94	51.16	144.18	129.60	84.04	179.37	<0.001

*Adj, adjusted*.

*Energy and nutrient requirements from complementary foods based on average breast milk consumption for children in the age groups 6–8, 9–11, and 12–23 months (57)*.

a *Estimated requirement for vitamin B_6_ from complementary foods for children in the age groups 6–8, 9–11 and 12–23 months = 0. Results represent data for non-breastfed children aged 6–23 months who consumed complementary foods during both seasons*.

b *Estimated requirements for folic acid and vitamin C from complementary foods for children in the age groups 6–8 and 9–11 months = 0. Results represent data for breastfed children aged 12–23 months and non-breastfed children aged 6–23 months who consumed complementary foods during both seasons*.

† *Median percentage met requirements for energy and other nutrients from complementary foods comparisons done only for children who received complementary foods during both seasons*.

* *Wilcoxon signed-rank test, used to determine differences in median percentage met requirements for energy and other nutrients from complementary foods between the 2 seasons. Adjusted P-values-Bonferroni–Holm correction test for multiple comparisons*.

### Seasonal Effects on Dietary Diversity of Women and Children

**Figure 3:**
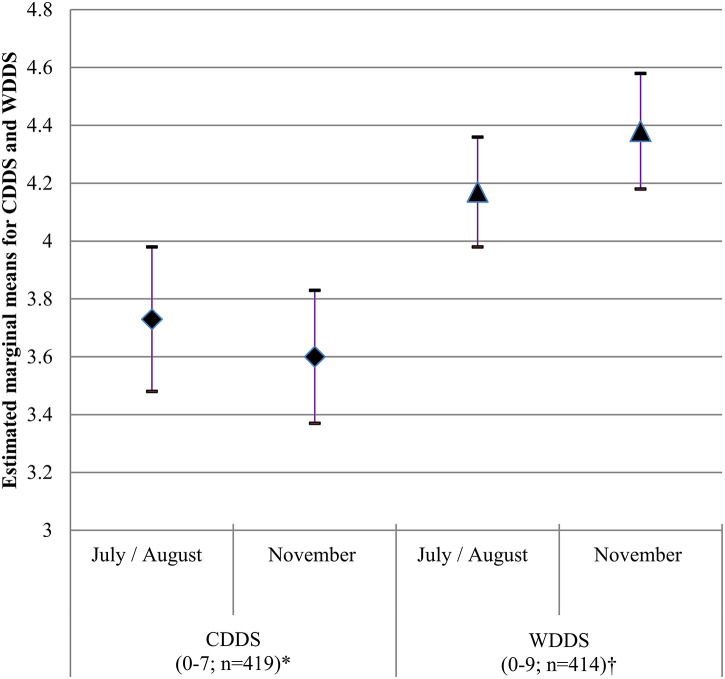
Estimated marginal means, 5th and 95th CI for WDDS (*n* = 414) and CDDS (*n* = 419) in July/August and November 2012 in Western Kenya. Covariates included in GENLINMIXED models: *age of women (years), education of women (years), wealth index, household size, household hunger scale score, home gardening, ethnic group, agro-ecological zone and sub-county, †age of children (months), wealth index, ethnic group, household size, household hunger scale score, home gardening, and agro-ecological zone and sub-county.

The results from the GENLINMIXED models with regard to the effect of seasonality on WDDS and CDDS are presented in Figure 3. Seasonality was found to have a small but significant effect on WDDS (*P*=0.008) but not on CDDS (*P* = 0.293). While the estimated marginal mean (SE) WDDS increased from 4.17 (0.10) in July/August to 4.38 (0.10) in November, estimated marginal mean CDDS (SE) decreased from 3.73 (0.13) to. 3.60 (0.11) during the 2 time points.

To better understand the above differences in DDS across the seasons, we further assessed the seasonal variations in DDS as a function of ethnic group. The seasonal effect was different for the women from the 3 ethnic groups (*P* interaction <0.001). The estimated marginal mean (SE) WDDS increased between July/August and November for Luhya [4.14 (0.12) vs. 4.79 (0.15)] and Teso [4.05 (0.20) vs. 4.17 (0.18)] women, and decreased for Luo women [4.33 (0.13) vs. 4.20 (0.16)] between the seasons (Table 6). The estimated marginal mean WDDS were significantly different among women from the 3 ethnic groups in November, *P* = 0.006. The same phenomenon was observed with regard to seasonal variations on CDDS as a function of ethnic group, *P* (interaction) <0.001. While the estimated marginal mean CDDS for Luo [3.93 (0.20) vs.3.40 (0.17)] and Teso [3.63 (0.31) vs. 3.51 (0.27)] children decreased, that for Luhya children [3.62 (0.17) vs. 3.91 (0.17)] increased between the seasons. However, the estimated marginal mean CDDS were not significantly different for the children from the 3 ethnic groups during each season.

**Table 6:** Effects of seasonality and other factors on WDDS and CDDS in Western Kenya.

**Variables**		***n***	**WDDS (*****n*** **=** **414)**^****a****^				**CDDS (*****n*** **=** **419)**^****b****^				
			**Estimated marginal mean**	**SE**	**95% Cl**	P^*^	***n***	**Estimated marginal mean**	**SE**	**95% Cl**	P^*^
Survey	July/August	414	4.17	0.10	3.98, 4.36	0.008	419	3.73	0.13	3.48, 3.98	0.293
	November	414	4.38	0.10	4.18, 4.58		419	3.60	0.11	3.39, 3.81	
Survey*ethnic group						<0.001					<0.001
July/August	Luo	110	4.33	0.13	4.08, 4.60	0.390	110	3.93	0.20	3.56, 4.33	0.592
	Luhya	229	4.14	0.12	3.90, 4.39		232	3.62	0.17	3.31, 3.96	
	Teso	75	4.05	0.20	3.68, 4.45		77	3.63	0.31	3.08, 4.29	
November	Luo	110	4.20	0.16	3.90, 4.52	0.006	110	3.40	0.17	3.09, 3.75	0.113
	Luhya	229	4.79	0.15	4.50, 5.09		232	3.91	0.17	3.59, 4.26	
	Teso	75	4.17	0.18	3.82, 4.55		77	3.51	0.27	3.03, 4.07	
Ethnic group	Luo	110	4.26	0.14	4.00, 4.54	0.195	110	3.66	0.16	3.35, 3.99	0.723
	Luhya	229	4.45	0.12	4.22, 4.70		232	3.76	0.16	3.47, 4.08	
	Teso	75	4.11	0.18	3.78, 4.47		77	3.57	0.26	3.10, 4.11	
Agro-ecological zones	UM1	101	4.44	0.21	4.06, 4.87	0.656	104	3.73	0.21	3.34, 4.18	0.659
	LM1	147	4.12	0.10	3.94, 4.32		147	3.47	0.15	3.19, 3.77	
	LM2	59	4.19	0.11	3.97, 4.41		61	3.60	0.18	3.26, 3.98	
	LM3	34	4.33	0.26	3.86, 4.87		34	3.62	0.27	3.12, 4.20	
	LM4	27	4.34	0.28	3.84, 4.92		27	3.97	0.30	3.43, 4.59	
	LM5	46	4.21	0.16	3.90, 4.54		46	3.60	0.25	3.14, 4.13	
Home gardening	Yes	332	4.33	0.09	4.17, 4.50	0.379	337	3.80	0.14	3.59, 3.82	0.044
	No	82	4.21	0.14	3.95, 4.49		82	3.53	0.10	3.60, 4.00	
Sub-county	Bondo	107	4.20	0.07	4.06, 4.35	0.190	107	3.66	0.09	3.49, 3.83	0.943
	Mumias	104	4.48	0.23	4.05, 4.97		105	3.68	0.27	3.18, 4.26	
	Teso	102	4.20	0.07	3.06, 4.35		103	3.66	0.09	3.49, 3.83	
	Vihiga	101	4.20	0.07	4.06, 4.35		104	3.66	0.09	3.49, 3.86	

*WDDS, women's dietary diversity score; CDDS, children's dietary diversity score; SE, standard error; CI, confidence interval*.

a *Covariates in model include wealth index, household size, age of women (years), education of women (years), household hunger score*.

b *Covariates in model include wealth index, age of children (months), household size, household hunger scale score*.

* *GENLINMIXED model test*.

**Figure 4:**
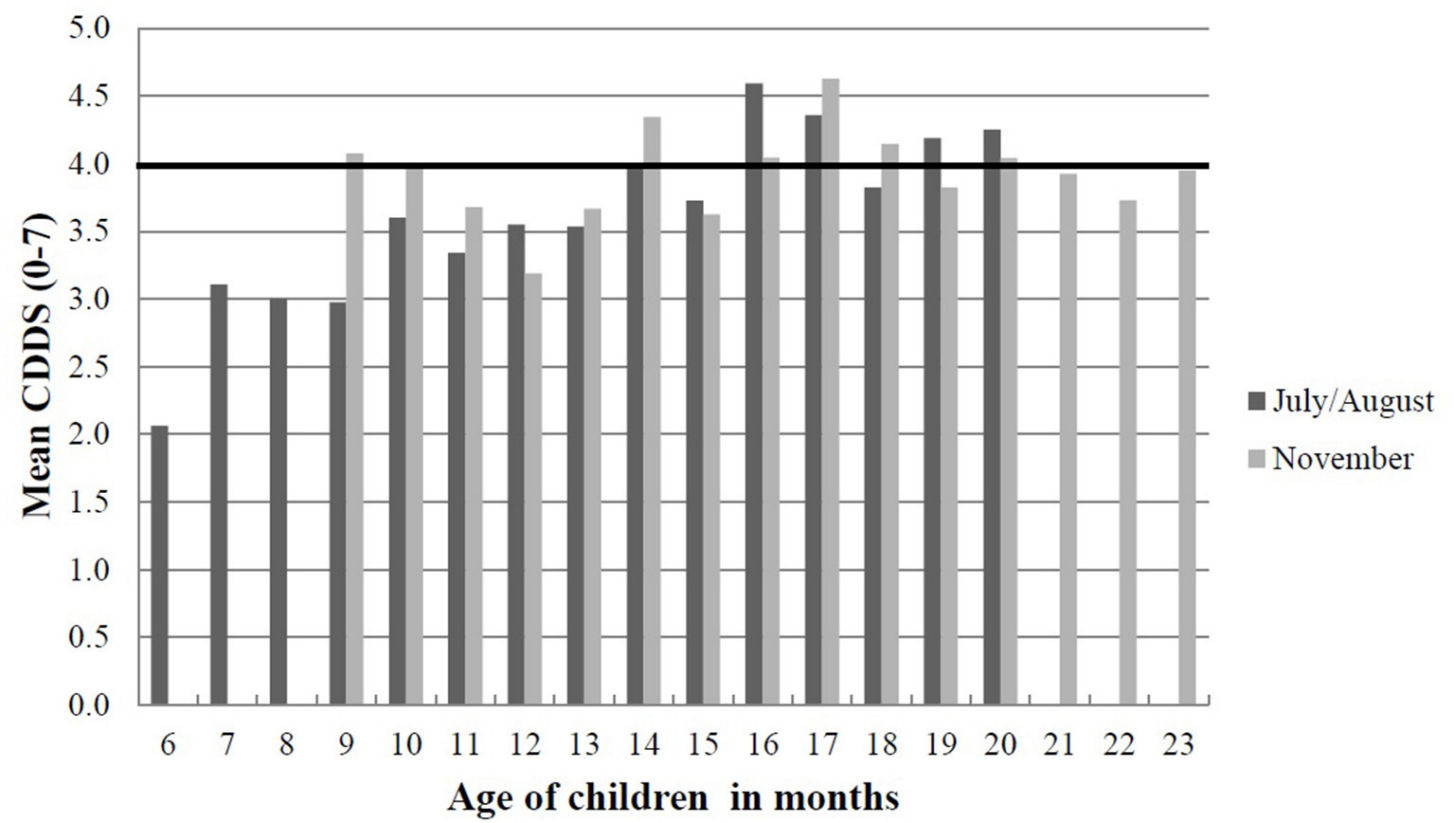
Mean CDDS of children below 2 years stratified by age in months in July/August and November 2012 in Western Kenya. The black horizontal line indicates the threshold for minimum dietary diversity that 6–23 months old children should achieve from their diets according to WHO (51). The data presented is from a cohort of children (*n* = 426) who were aged 6–20 months in July/August and 9–23 months in November; thus, there were no children included aged 21–23 months in July/August and 6–9 months in November. CDDS, children's dietary diversity score.

We further calculated the mean CDDS of the children stratified by age in July/August and November to confirm the results with regard to the lack of a seasonal effect on CDDS. The results did not show a clear trend in the distribution of CDDS among the children between the two seasons. However, older children had higher mean CDDS compared to younger children during each season (Figure 4).

### Factors Associated With Dietary Diversity and Nutrient Intake

Results from the GENLIMIXED models showed that education of women (years) had a small but positive effect on both WDDS [odds ratio (OR) = 1.01 95% CI 1.00, 1.01, *P* = 0.022] and CDDS (OR= 1.01 95% CI 1.00, 1.02, *P* = 0.005) (Table 7). On the other hand, household food insecurity had a negative influence on both WDDS (OR = 0.95 95% CI 0.93, 0.96, *P* < 0.001) and CDDS (OR = 0.94 95% CI 0.92, 0.96, *P* < 0.001). While increasing age (in years) among women had a very small negative effect on WDDS (OR = 1.00 95% CI 0.99, 1.00, *P* = 0.033), increasing age (in months) among children had a positive effect on CDDS (OR = 1.02 95% CI 1.01, 1.02, *P* < 0.001).

**Table 7:** Factors associated with dietary diversity among women and children in Western Kenya (*n* = 414).

	**WDDS (*****n*** **=** **414)**^****a****^				P*	**CDDS (*****n*** **=** **414)**^****b****^				P^*^
	**Coefficient**	**SE**	**OR**	**95% CI**		**Coefficient**	**SE**	**OR**	**95% CI**	
Education of women (years)	0.01	0.00	1.01	1.00, 1.01	0.022	0.01	0.00	1.01	1.00, 1.02	0.005
Household food insecurity	−0.06	0.01	0.95	0.93, 0.96	<0.001	−0.06	0.03	0.94	0.92, 0.96	<0.001
Age of women (years)	−0.01	0.02	1.00	0.99, 1.00	0.033	−0.00	0.00	1.00	0.99, 1.00	0.070
Household size	0.00	0.00	1.00	0.99, 1.01	0.978	0.01	0.01	1.01	0.99, 1.02	0.458
Wealth index	0.00	0.00	1.00	1.00, 1.01	0.350	0.00	0.01	1.00	0.99, 1.01	0.640
Age of children (months)						0.01	0.00	1.02	1.01, 1.02	<0.001

*WDDS, women dietary diversity score; CDDS, children's dietary diversity score; SE, standard error; OR, odds ratio; CI, confidence interval*.

a *Covariates in model: ethnic group, home gardening, agro-ecological zone, and sub-county*.

b *Covariates in model: ethnic group, home gardening, agro-ecological zone, and sub-county*.

* *GENLINMIXED model test*.

Results from the UNIANOVA analyses, showed that agro-ecological zones influenced the intakes of energy (*P* = 0.015), carbohydrates (*P* = 0.005), protein (*P* < 0.001), vitamin A (*P* = 0.013), iron (*P* < 0.001), zinc (*P* = 0.003), and calcium (*P* < 0.001), with a trend of higher nutrient intakes among women residing in the semi-humid lower midlands zone (LM3) and lower intakes among those living in the humid upper midland zones (UM1) (Table 8). We also found significant associations between ethnic group and intakes of energy (*P* = 0.015) and fat (*P* = 0.005) with higher intakes among Luhya women compared with those from the Luo and Teso ethnic groups. Additional results from the same UNIANOVA models showed that household food insecurity had a negative effect on the intakes of energy (*P* = 0.046), carbohydrates (*P* = 0.046), protein (*P* = 0.026), and calcium (*P* < 0.001) (Table 8). Home gardening had a positive influence on the intakes of carbohydrate (*P* = 0.025), iron (*P* = 0.045), and zinc (*P* = 0.041). While higher age had a positive effect on vitamin A intake (*P* = 0.005), increasing household size had a negative influence on vitamin A intake (*P* = 0.010).

**Table 8:** Factors influencing nutrient intakes among women in Western Kenya (*n* = 404).

**Nutrients**		**B coefficient**	**SE**	P^*^	**95 % CI**
Energy, kJ^a^	Wealth index	−27.17	56.98	0.634	−139.21, 84.88
	Women's age (years)	8.35	18.94	0.660	−28.89, 45.57
	Education (years)	−34.33	42.56	0.420	−118.01, 49.35
	Household food insecurity	−217.50	108.58	0.046	−430.99, −4.01
	Breastfeeding status	−63.17	381.23	0.868	−812.76, 686.43
	Household size	−4.73	64.43	0.942	−131.40, 121.95
	Home gardening	651.14	344.42	0.059	−26.09, 1,328.37
Protein, g^b^	Wealth index	−0.10	0.56	0.855	−1.20, 1.00
	Women's age (years)	−0.18	0.17	0.326	−0.55, 0.18
	Education (years)	−0.02	0.42	0.964	−0.84, 0.80
	Household food insecurity	−2.39	1.07	0.026	−4.49, −0.29
	Breastfeeding status	3.77	3.75	0.314	−3.59, 11.14
	Household size	0.15	0.63	0.812	−1.09, 1.40
	Home gardening	5.12	3.39	0.131	−1.54, 11.77
Fat, g^c^†^^	Wealth index	0.01	0.01	0.398	−0.01, 0.04
	Women's age (years)	−0.00	0.00	0.394	−0.01, 0.01
	Education (years)	0.01	0.01	0.530	−0.01, 0.02
	Household food insecurity	−0.04	0.02	0.106	−0.09, 0.01
	Breastfeeding status	0.08	0.08	0.313	−0.08, 0.25
	Household size	−0.01	0.01	0.381	−0.04, 0.02
	Home gardening	0.05	0.08	0.511	−0.10, 0.20
Carbohydrates, g^d^	Wealth index	−3.21	2.57	0.212	−8.27, 1.84
	Women's age (years)	1.10	0.85	0.197	–.58, 2.78
	Education (years)	−2.93	1.92	0.128	−6.71, 0.84
	Household food insecurity	−9.82	4.90	0.046	−19.45, −0.19
	Breastfeeding status	−17.52	17.20	0.309	−51.33, 16.30
	Household size	1.19	2.91	0.682	−4.53, 6.91
	Home gardening	35.00	15.54	0.025	4.45, 65.55
Vitamin A, μg^e^	Wealth index	18.05	13.79	0.191	−9.05, 45.16
	Women's age (years)	13.11	4.60	0.005	4.07, 22.15
	Education (years)	−9.16	10.29	0.374	−29.39, 11.07
	Household food insecurity	1.39	26.29	0.958	−50.31, 53.09
	Breastfeeding status	−32.24	92.14	0.727	−213.42, 148.94
	Household size	−40.96	15.80	0.010	−72.02, −9.90
	Home gardening	156.92	83.28	0.060	−6.84, 320.68
Iron, mg^f^	Wealth index	−0.09	0.13	0.473	−0.35, 0.16
	Women's age (years)	−0.01	0.04	0.758	−0.10, 0.07
	Education (years)	−0.15	0.10	0.119	−0.34, 0.04
	Household food insecurity	−0.32	0.25	0.191	−0.80, 0.16
	Breastfeeding status	−0.12	0.86	0.889	−1.81, 1.57
	Household size	−0.01	0.15	0.975	−0.29, 0.28
	Home gardening	1.57	0.78	0.045	0.04, 3.10
Calcium, mg^g^	Wealth index	0.55	5.07	0.913	−9.42, 10.53
	Women's age (years)	2.31	1.69	0.171	−1.01, 5.62
	Education (years)	−2.39	3.79	0.530	−9.84, 5.07
	Household food insecurity	−34.13	9.67	<0.001	−53.13, −15.12
	Breastfeeding status	−1.26	33.94	0.970	−67.10, 65.48
	Household size	−0.05	5.74	0.993	−11.33, 11.23
	Home gardening	42.71	30.67	0.165	−17.59, 103.0
Zinc, mg^h^	Wealth index	−0.13	0.09	0.148	−0.30, 0.05
	Women's age (years)	−0.01	0.03	0.779	−0.07, 0.05
	Education (years)	0.00	0.07	0.981	−0.13, 0.13
	Household food insecurity	−0.30	0.17	0.070	−0.63, 0.025
	Breastfeeding status	0.29	0.58	0.623	−0.86, 1.43
	Household size	0.03	0.10	0.743	−0.16, 0.23
	Home gardening	1.08	0.53	0.041	0.05, 2.12

*SE, standard error; CI, confidence interval*.

a *R^2^ = 0.089 (adjusted R^2^ = 0.055)*,

b *R^2^ = 0.117 (adjusted R^2^ = 0.084)*,

c *R^2^ = 0.092 (adjusted R^2^ = 0.058)*,

d *R^2^ = 0.104 (adjusted R^2^ = 0.070)*,

e *R^2^ = 0.105 (adjusted R^2^ = 0.072)*,

f *R^2^ = 0.114 (adjusted R^2^ = 0.081)*,

g *R^2^ = 0.183 (adjusted R^2^ = 0.153)*,

h *R^2^ = 0.085 (adjusted R^2^ = 0.051)*.

† *Variable log-transformed before analysis*.

* *UNIANOVA test*.

*Covariates in models include agro-ecological zone and ethnic group*.

### Association Between WDDS and CDDS

We performed additional analyses using Pearson's correlation tests to cheque for relationships between WDDS and CDDS in both July/August and November, and also between change in WDDS and change in CDDS between the seasons. WDDS was significantly correlated with CDDS both in July/August (*r* = 0.39, *P* < 0.001) and November (*r* = 0.45, *P* < 0.001). Additionally, there was a significant relationship between change in WDDS and change in CDDS between the seasons (*r* = 0.32, *P* < 0.001).

## Discussion

In this study WDDS was found to be sensitive to seasonal changes, with the scores being higher in November (post-harvest season) compared with July/August (pre-harvest season). Similar findings of seasonal variations in DDS among women during different seasons have been reported in other studies conducted in rural areas in developing countries (12, 13, 21, 35, 61). The observed increased consumption of legumes, nuts and seeds by women during the post-harvest season in November could be attributed to increased availability following the annual harvest. This result indicate that seasonality can contribute to variations in farm production diversity and consequently variations in the quality of diets consumed among subsistence farm households. In a study conducted in rural Nigeria, no relationship was observed between farm production diversification and DD during the post-planting season, pointing to the existence of seasonal consumption poverty reflected by a decline in DD among rural households (32). Consumption of diets low in diversity is a common occurrence in many countries in sub-Saharan Africa and is linked to chronic shortages of vegetables and fruit crops during the dry seasons (62). In the current study, the short rains experienced during the post-harvest season could explain the observed increase in consumption of dark green leafy vegetables, which depend on either rain or irrigation and were thus readily available during this seasons (63). Further, in many developing countries, Kenya included, it is common practise for small holder farmers to sell their surplus food crops to the market immediately following the harvest time, often at very low prices. Unfortunately, this period of cheap food supply following the harvest time is usually followed by food purchases at high prices during the lean or planting time. The seasonal price instability experienced during the lean season negatively affects DD for many small-holder farmers who also have limited financial resources (64). While agro-ecological potential did not have a significant effect on the DD of women and children in this study, on the contrary in South Africa, household DD declined with decreasing agro-ecological potential from the wet to the drier areas with better agro-ecological potential favouring agriculture and reducing dependence on food purchasing (65). This points to the need for rural households to allocate more to land to subsistence farming in order to not only diversify food access through production of their own foods but also lower their dependence on food purchasing especially during the lean season. In addition to growing crops adapted to such seasons, the use of wild foods which are excellent sources of nutrients and contribute to reduced diet costs while filling nutrient gaps year-round, need to be promoted. For example, in Turkana, Kenya, including 3 wild fruits and 3 wild vegetables which were available throughout the seasons contributed to improved diets for women and children (66). Decline in DD and reduced ability to maintain the same level of nutrition quality during the off-seasons could also be attributed to lack of proper storage facilities, particularly for perishable foods in rural areas (67). Interventions to promote simple innovations to enhance preservation of perishable surplus foods following the annual harvest in order to extend their shelf life could help in covering seasonal gaps in food availability and accessibility. This could consequently enhance overall household food security and maintain DD across seasons among resource limited smallholder rural farm households.

Another key issue that we assessed in this study is the seasonal variation in nutrient intakes among women and children aged 6–23 months. The intake of energy and most nutrients were slightly higher among the women during the post-harvest season in November. Except for vitamin E, iron, and calcium intakes which were significantly different between the seasons, the intake of energy and other nutrients did not differ significantly among the women between the seasons. Similar results were found in a study conducted among women in rural Burkina Faso (33). The observed increase in the intakes of vitamin E, iron and calcium by the women in November could be partly explained by the overall increase in the consumption of foods from more food groups during this season. The fact that no seasonal effects were observed in the intakes of energy and other nutrients (except for iron, calcium and vitamin E) alludes to the fact that, households are able to adapt and change their feeding patterns to a certain extent in order to cope during periods of food shortage. This is at least true for energy intake from staple foods which are usually given priority in production, buying and consumption. In this study, the amounts of different foods (g/day) consumed by the women were not significantly different between the seasons, a fact that could also partly explain the lack of seasonal differences in the intakes of energy and most nutrients. This finding suggests that while an increase in food availability during the harvest season may contribute to improved DD, it may not necessarily lead to improved energy and nutrient intakes if the actual amounts of different foods consumed are inadequate to meet the nutrient requirements for the different population groups. Thus, interventions seeking to improve the consumption of a variety of foods should also include strategies to promote the consumption of adequate amounts of different foods to ensure overall adequate nutrient intakes for the different population groups.

Overall, inadequate nutrient intake among the women, i.e., not meeting the EAR, was found for most nutrients during both seasons. Similar findings of inadequate nutrient intakes among women have been reported in other studies conducted in Kenya (68, 69), South Africa (70), Burkina Faso (71), and Bangladesh (72), providing evidence of generally low nutrient intakes among women, and which is a reflection of overall poor quality diets across seasons. The observed changes in the intakes of key nutrients such as iron and calcium during the post-harvest season in this study indicate that in most cases, families in rural areas tend to subsist only on a subset of foods mainly from their own production. In many cases, these families could have access to a variety of other local nutrient-rich foods, but these alternative foods are often abandoned in favour of a limited number of foods, usually the energy-dense staples (9). This reliance on a limited number of foods in the presence of a variety of other different local foods could be attributed to many factors including lack of access due to high prices, low production, cultural food habits and practises, inadequate nutrition knowledge and skills on the available local foods and how to utilise them for improved diet quality (73–75). Therefore, in addition to behaviour change communication strategies to promote the consumption of a variety of foods across seasons, there is need for agricultural strategies to ensure that farmers produce a variety of foods that can substitute each other and are nutritionally appropriate during different seasons.

In this study the children also consumed foods from more food groups during the post-harvest season compared to the harvest season. However, seasonality was found not to have an effect on CDDS. This finding is explained by the fact that the children had grown older during the post-harvest season, and were thus more likely to be fed on a variety of foods (76, 77). Nevertheless, the diets of the children comprised mainly of cereal-based starchy staples with low consumption of animal source products during both seasons, a common practise that has also been reported in other studies conducted in developing countries (17, 78–80). In addition, the share of energy and nutrient requirements met from complementary foods was higher in November compared with July/August, also confirming the effect of age, with older children receiving more nutrients from complementary foods. The fact that seasonality did not have an effect on CDDS indicates that while there may be variations in food availability across seasons, in most cases foods fed to young children during the complementary feeding period do not change to an extent of influencing their DD. This indicates that other factors than food availability exerts influences on the quality of diets fed to young children, particularly during the complementary feeding period. In the present study the effect of seasonality on DD was assessed during the harvest and post-harvest seasons. More seasonal variations in dietary patterns should be expected as the observed seasons were not representative of the year-round food availability calendar. However, for children under 2 years, the age effect seems to be more important. However, it should be expected that with increasing age, reduced breastfeeding and more reliance on family foods, young children would also be affected by seasonality once they grow out of the complementary feeding period. There is therefore need for behaviour change interventions to sensitise caregivers on the importance of feeding young children a variety of family foods, including animal source foods during the complementary feeding during all seasons.

The finding that WDDS and CDDS were positively associated is consistent with those reported from other studies (76, 81). While this may not be surprising, yet, it also cannot be assumed automatically as it is suggested by an increasing number of double burden households with overweight/obese mothers and underweight or stunted children (82). While it is expected that women and children from households having access to a variety of foods should consume better quality diets, this is not always the case. Analysis of data from the 2008 Ghana Demographic and Health Survey showed that not all the foods consumed by the mothers were given to the children (83). This suggests that in most cases caregivers usually tend to rely only on a subset of family foods when feeding young children due to several factors including inadequate nutrition knowledge, cultural practises and beliefs and limited time for child care (74, 84). Still, this finding indicates that maternal diets may be an important determinant of children's diets. Therefore, interventions aimed at improving the quality of diets consumed by children should include strategies to also promote maternal diets as this may have a direct effect on the diets consumed by other family members, including the children.

Ethnic group was found to have an effect on both DD and nutrient intake among the women. Ethnicity influences dietary habits with different ethnic groups ascribing to different traditional food cultures, which influence food consumption patterns including food choices and preferences (85, 86). The different food beliefs and taboos also influence the consumption of certain foods, particularly animal source foods, and which in most cases are tilted in favour of men (87), consequently affecting the quality of diets consumed by women and children. The effects of ethnicity on food patterns have been reported in other studies (61, 85, 88). The evaluation of behavioural strategies of population groups to improve nutrition status have been recently identified as key research priorities (89), which need to be implemented. Such strategies would help in the identification of specific cultural barriers that are a hindrance to the consumption of specific foods among population groups, which should be addressed in order to promote good nutrition.

Socio-demographic factors including maternal education and food security were found to have a positive effect on the women and children DDS. Older age among the children was associated with higher DDS. Socio- demographic factors and household food security have been shown to be associated with dietary patterns of women and children in a similar way in other studies (76, 90, 91). However, socio-economic status assessed using the wealth index was not found to have a significant influence on DD and nutrient intake in this study. Strategies to improve dietary intakes for both women and children should therefore also consider incorporating actions to address some of the underlying constraints including low levels of education among the women and household food insecurity, which would in the long run lead to improvements in the dietary intakes of all household members, and in particular of the women and children.

## Strengths and Limitations of the Study

The strength of this study are the repeated cross-sectional surveys conducted during different seasons within the same years and targeting the same woman and their children. This enabled us to assess not only the effect of seasonality on DD of women and their children aged 6–23 months, but also the seasonal differences in their food (g/day) and nutrient intakes. Even though the seasons were not very distinct (July/August-harvest and November-post-harvest), our results still showed a small but significant seasonal effect on WDDS as well as seasonal variations in vitamin E, iron and calcium intakes among the women. Anthropometric measurements for the study participants were taken only once during the survey in July/August, thus, we were not able to assess the effects of seasonality on nutritional status of the women, which would have enriched the study. Future similar studies should be conducted during more distinct seasons in terms of food availability such as during the pre-harvest and harvest seasons that would clearly show the effects of seasonality on both dietary intakes and nutritional status outcomes. In addition, similar studies should be conducted with data collected at more time points during the same year and during different years, as this could be more informative.

A limitation of our study was attributed to the use of a single 24-h recall to assess dietary intakes of the study participants during each season. Like other dietary recall methods the 24-h recall method relies on memory in terms of identification of the foods eaten as well as the estimation of food portions consumed (92). To reduce this type of error, we used highly trained enumerators who spoke the local languages during the interviews with the women. We also used local household measures to estimate the amounts of foods consumed by the women and the children (93). On the other hand, to recall the last 24-h only is much more accurate than longer periods such as the last week or even month. The use of single 24-h recall may not be representative for estimating the individual's usual nutrient intake due to variability in the day to day intakes of foods (40). The 24-h recall is also prone to the problems of under-reporting and over-reporting and whether or not to exclude cases with extreme energy intake values is debatable.

In addition to assessing the effect of seasonality on women's DD, this study also analysed the seasonal differences in nutrient intakes of the women. We decided in favour of excluding women who had unusual low and high energy intakes during any of the two surveys because extreme intakes on any 1 day (which were expected due to sickness or festivities) would have led to a misinterpretation of the results, with regards to seasonal food availability. This may have introduced bias and limited our findings, which may not be representative of usual food intakes among the women in the target population. Despite these limitations, this study contributes to the existing, yet, little literature on the effects of seasonality on DD and nutrient intake in rural Kenya.

## Conclusion

The results from this study showed that seasonality had an effect on the quality of diets consumed by women in rural households. Different to the women, the diets of the children were not affected by seasonality, indicating that other factors apart from food availability exert an influence on the dietary patterns of young children during the complementary feeding period. Still, it should be expected that as the children grow older and transit from the complementary feeding period to rely on family foods, their diets would also be affected by seasonality. Thus, integrated programs aimed at strengthening rural households' resilience against seasonal deterioration in diet quality are recommended. Seasonality should also be included as a component in nutrition education programs not only as a way to promote behaviour changes and increase nutrition knowledge among caregivers, but also to ensure that households have access to and utilise a variety of foods during all seasons. This could include promotion of preservation of vegetables and fruits to cover periods with limited access to fresh vegetables and fruits. The behaviour change strategies should also focus their key messages on addressing cultural barriers hindering the consumption of a variety of foods among vulnerable population groups including women and young children throughout the year. The strategies should promote the consumption of local foods that are culturally acceptable, affordable, and nutritious during all seasons, which could contribute to improvements in the overall quality of women's and children's diets.

## Data Availability Statement

The raw data supporting the conclusions of this article will be made available by the authors, without undue reservation.

## Ethics Statement

The studies involving human participants were reviewed and approved by National Council of Science and Technology (NCST) Nairobi, Kenya. Written informed consent to participate in this study was provided by the participants' legal guardian/next of kin.

## Author Contributions

LW was responsible for data collection, statistical analysis and manuscript preparation with contributions from GK, IJ, and MK. GK was the principle investigator and contributed to the conceptualisation of the study design. All the authors read and approved the final manuscript.

## Conflict of Interest

The authors declare that the research was conducted in the absence of any commercial or financial relationships that could be construed as a potential conflict of interest.
